# TMIGD1: Emerging functions of a tumor supressor and adhesion receptor

**DOI:** 10.1038/s41388-023-02696-5

**Published:** 2023-04-22

**Authors:** Eva-Maria Thüring, Christian Hartmann, Ysabel A. Schwietzer, Klaus Ebnet

**Affiliations:** Institute-associated Research Group “Cell adhesion and cell polarity”, Institute of Medical Biochemistry, ZMBE, University of Münster, Münster, Germany

**Keywords:** Cell adhesion, Cancer

## Abstract

The development of multicellular organisms depends on cell adhesion molecules (CAMs) that connect cells to build tissues. The immunoglobulin superfamily (IgSF) constitutes one of the largest families of CAMs. Members of this family regulate such diverse processes like synapse formation, spermatogenesis, leukocyte-endothelial interactions, or epithelial cell-cell adhesion. Through their extracellular domains, they undergo homophilic and heterophilic interactions in cis and trans. Their cytoplasmic domains frequently bind scaffolding proteins to assemble signaling complexes. Transmembrane and immunoglobulin domain-containing protein 1 (TMIGD1) is a IgSF member with two Ig-like domains and a short cytoplasmic tail that contains a PDZ domain-binding motif. Recent observations indicate that TMIGD1 has pleiotropic functions in epithelial cells and has a critical role in suppressing malignant cell behavior. Here, we review the molecular characteristics of TMIGD1, its interaction with cytoplasmic scaffolding proteins, the regulation of its expression, and its downregulation in colorectal and renal cancers.

## Introduction

Epithelia consist of sheets of cells in which cells are connected by intercelluar junctions. Individual cells are highly polarized with an apical membrane domain facing the cell-free outside of an organ, a lateral domain contacting the adjacent cell, and a basal membrane domain that is attached to the underlying extracellular matrix [1]. This organization is commonly referred to as apico-basal polarity [2]. A loss of apico-basal polarity does not only impair the functioning of the individual cell but is frequently associated with malignant growth [1]. A loss of apico-basal polarity is also frequently associated with a loss of cell-cell adhesion and with a transition from an epithelial phenotype to a mesenchymal phenotype, thus predisposing cells to dissemination and metastasis formation [3–5].

The intercellular adhesion of epithelial cells is mediated by different cell adhesion receptors, in particular cell adhesion receptors of the cadherin and of the immunoglobulin (Ig) superfamilies (SF). Many adhesion receptors are incorporated into structural networks at specific membrane domains like adherens junctions (AJs), tight junctions (TJs) or desmosomes [6]. A common feature of these adhesive networks is their association with the actin cytoskeleton or the intermediate filament system through direct or indirect interacions of adhesion receptors with cytoplasmic scaffolding proteins [7]. Another commonality of adhesive networks is an extensive cross-talk with other adhesive structures, both at sites of cell-cell adhesion and at sites of cell-matrix adhesion [8–11]. This enables cells to integrate signals originating from different adhesive sites, and to transform these signals into coordinated cell behavior, as it is required during collective cell migration or during morphogenesis [12, 13]. Thus, cell-cell adhesion receptor-based structures not only provide mechanical links between individual cells but represent critical signaling networks that orchestrate cell behavior at the tissue scale.

Given the critical role of cell-cell adhesion receptors in maintaining tissue integrity both by their adhesive function and by their signaling function, it is not surprising that the expression levels of cell-cell adhesion receptors are frequently altered in malignancies. For example, during epithelial-to-mesenchymal transition (EMT), genes encoding adhesion receptors including E-cadherin, claudins, or Crumbs3, and their cytoplasmic binding partners including ZO-1, Pals1, PATJ, or plakophilin are actively repressed by SNAIL, bHLH or ZEB transcription factors [4]. Other adhesion receptors including Epithelial Cell Adhesion Molecule (EpCAM) are overepressed in some tumors but downregulated in others indicating that both increased and decreased expression of a given cell-cell adhesion receptor can contribute to tumor formation, which points to a tumor context-specific function [14, 15].

The IgSF member transmembrane and immunoglobulin domain-containing protein 1 (TMIGD1) is predominantly expressed by intestinal and renal epithelial cells. It has been identified on the basis of a striking progressive downregulation during the development of colorectal cancer [16]. Based on recent findings it has become clear that TMIGD1 has pleiotropic functions, including the regulation of cell proliferation, cell migration, mitochondrial activity and brush border assembly. In this review article, we summarize the current knowledge of its biology. We describe its structural organization as an adhesion receptor, its association with cytoplasmic binding partners, the regulation of its expression, and its downregulation in colorectal and renal cancer.

## TMIGD1 as adhesion receptor

**Fig. 1:**
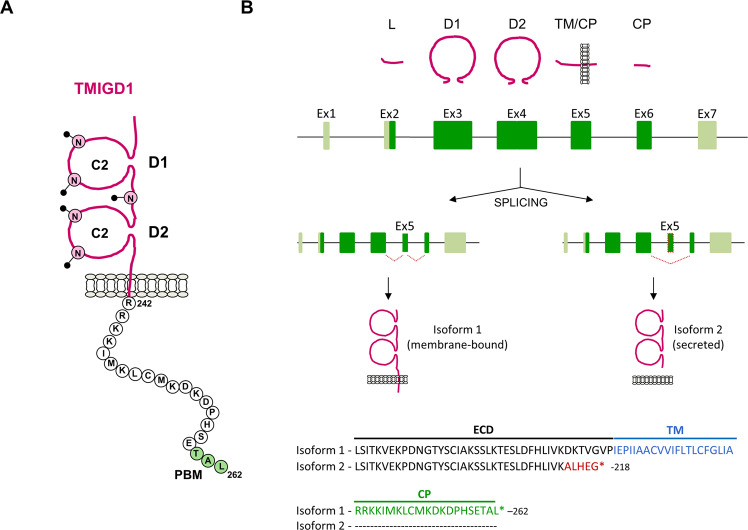
TMIGD1: Principal organization of the protein and genomic organization. **A** Organization of human TMIGD1. The two Ig-like domains are indicated by D1 (membrane-distal, V_30_ - S_114_, C2-type) and D2 (membrane-proximal, P_122_ - D_207_, C2 type). Disulfide bridges involve C_54_ - C_103_ (D1) and C_143_ - C_195_ (D2). The five potential N-glycosylation sites (N-glycans, N_58_, N_83_, N_118_, N_158_, N_190_) are indicated by symbols (filled circles). The type I PDZ domain-binding motif (PBM, T_260_A_261_L_262_) is highlighted in green. Amino acids of the cytoplasmic domain are depicted as single letter code. **B** Genomic organization of the human TMIGD1 gene. The TMIGD1 gene consists of seven exons. Coding regions are indicated in green, non-conding regions are depicted in lime-green. The TMIGD1 protein segments encoded by the five coding exons are depicted in magenta. Alternative splicing results in skipping of exon 5 and, as a consequence in a premature Stop codon, generating a secreted version of TMIGD1 (isoform 2). The AA sequence shown at the bottom starts with L_180_. The five AA and the premature Stop codon arising from alternative splicing in isoform 2 are depicted in red. L leader peptide, D1 D1 Ig-like domain, D2 D2 Ig-like domain, ECD extracellular domain, Ex Exon, TM transmembrane, CP cytoplasmic.

TMIGD1 is a member of the Ig superfamily (IgSF) with two C2-type Ig-like domains, a single transmembrane domain and a short cytoplasmic domain consisting of 21 amino acids (AA) (Fig. 1A) [17]. The gene encoding TMIGD1 (human Gene ID: 388364, murine Gene ID: 66601) contains seven exons, two of which are non-coding exons (Fig. 1B). The five coding exons encode functionally distinct regions of the protein, such as the signal peptide, the two Ig-like domains, the transmembrane region, or the majority of the cytoplasmic region (Fig. 1B). Notably, exon 5 can be skipped by alternative splicing resulting in a protein that lacks the entire transmembrane domain and that represents a secreted version of TMIGD1 (Isoform 2, https://www.uniprot.org/uniprotkb/Q6UXZ0/entry, Fig. 1B). Based on the Human Protein Atlas database (https://www.proteinatlas.org/ENSG00000182271-TMIGD1/tissue), TMIGD1 mRNA is predominantly expressed in the gastrointestinal tract, including small intestine, colon and rectum, as well as in the kidney. In the gastrointestinal tract, TMIGD1 protein is expressed by differentiated cells of intestinal villi and of upper regions of colonic crypts [16, 18, 19]. In the kidney, TMIGD1 protein expression is restricted to proximal tubules and is absent from epithelial cells of distal tubules or by glomerular podocytes [20–22]. TMIGD1 protein expression has also been found in mesothelial cells lining the peritoneum [23]. In almost all other tissues TMIGD1 mRNA or protein expression is hardly detectable (https://www.proteinatlas.org/ENSG00000182271-TMIGD1/tissue) [20]. Also, in many cultured cell lines derived from different tissues, TMIGD1 expression is very low [16].

### The extracellular domain of TMIGD1

The extracellular domain of human TMIGD1 contains five potential N-glycosylation sites (Fig. 1A). These are located in the membrane-distal (D1) Ig-like domain (N58, N83), in the linker region between the two Ig-like domains (N118), and in the membrane-proximal (D2) Ig-like dormain (N158, N190) (Fig. 1A). Mutation analysis addressing the glycosylation of each of the five potential sites is still lacking, but it is likely that several of these sites are glycosylated in cells. Treatment of cellular lysates derived from TMIGD1-transfected cells with PNGase F, a glycosidase that removes N-linked oligosaccharides from glycoproteins, results in a shift of the relative molecular weight (M_R_) of TMIGD1 from ~43 kDa to ~26–27 kDa, which corresponds to the molecular weight of the mature TMIGD1 protein in the absence of posttranslational modifications (25.8 kDa) [16, 24, 25]. Removing the D1 domain results in hyperglycosylation which is completely lost after treatment with PNGase F, indicating that one or more of the three N-linked glycosylation sites in the linker region and the D2 domain are N-glycosylated [25]. Also, treatment of cultured cells with tunicamycin, an inhibitor of N-linked glycosylation in glycoprotein synthesis, results in a shift of the M_R_ of TMIGD1 to lower molecular weight species and a complete loss of the 43 kDa TMIGD1 species present in untreated cells [25]. Thus, TMIGD1 is exclusively N-glycosyslated.

Many members of the Ig-superfamily serve as homophilic cell-cell adhesion receptors [26]. Based on various observations, this applies for TMIGD1 as well. For example, a recombinant protein consisting of the recombinant extracellular region of TMIGD1 fused to GST can pulldown TMIGD1 from transfected cells [24], and similarly, a TMIGD1 construct that lacks the cytoplasmic domain co-immunoprecipitates with TMIGD1 [21]. In addition, transfected TMIGD1 promotes cell aggregation in non-aggregating cells [21, 24], and a recombinant TMIGD1 extracellular domain construct immobilized on beads promotes bead aggregation [21]. These observations strongly suggest that TMIGD1 acts as a homophilic adhesion receptor which supports cell-cell adhesion.

### The cytoplasmic domain of TMIGD1

The cytoplasmic domain of TMIGD1 consists of 21 AA. A prominent structural feature is a class I PDZ domain-binding motif (PBM) at the C-terminus [27]. PBMs are short linear peptide regions that in most cases are located at the C-termini of natural proteins [28] and that interact with PDZ domains through a canonical interaction that involves the carboxylate-binding loop of PDZ domains [28]. The presence of this motif suggests that TMIGD1 interacts with PDZ domain-containing scaffolding proteins. In fact, several PDZ domain-containing proteins have been identified as TMIGD1 binding partners. The functional relevance of these interactions will be discussed in detail below.

## TMIGD1 interaction partner and cellular functions

### SYNJ2BP

Synaptojanin-2-binding protein (SYNJ2BP) is a short protein that consists of a PDZ domain and a short C-terminal transmembrane segment through which it is inserted in the outer membrane of mitochondria [29]. It belongs to the group of tail-anchored (TA) proteins, which are inserted in the membranes of organelles and which are frequently involved in the targeting of organelles to the ER [30]. The PDZ domain of SYNJ2BP faces the cytoplasm and interacts with ribosome-binding protein 1 (RRBP1) localized in the ER membrane. SYNJ2BP, thus, supports the tethering of mitochondria to the ER [31]. SYNJ2BP is expressed in three isoforms, two of which lack the transmembrane segment and thus are not immobilized on mitochondria [32]. SYNJ2BP has also been found to interact with proteins localized in the plasma membrane and to regulate their endocytosis and signaling functions. These include the activin type II receptors [33] and the Notch ligands Delta like protein (DLL) 1 and DLL4 [34]. SYNJ2BP, thus, has diverse functions, and these functions are most likely dependent on its localization.

TMIGD1 directly interacts with SYNJ2BP through an interaction that involves the PBM of TMIGD1 and the PDZ domain of SYNJ2BP [25]. Interestingly, TMIGD1 localizes to mitochondria in kidney epithelial cells when cells are grown under sparse conditions, i.e., in the absence of homotypic cell contacts, and it gradually localizes to cell-cell junctions when cells are grown at higher cell densities [25]. The interaction with SYNJ2BP, therefore, likely serves to recruit TMIGD1 to mitochondria when cells are grown under sparse conditions. Notably, oxidative stress induces a degradation of TMIGD1 by the proteasome, and experimental manipulations of TMIGD1 expression showed a positive correlation between TMIGD1 expression levels and cell survival in the presence of oxidative stress [23, 24], which suggests a protective function of TMIGD1 during oxidative stress. Given that mitochondria are the major source of reactive oxygen species (ROS) like superoxide and hydrogen peroxide [35], the recruitment of TMIGD1 to mitochondria by SYNJ2BP suggests that TMIGD1 exerts a regulatory function during the generation of ROS directly at mitochondria. Since the generation of ROS is not principally harmful to cells (beneficial oxidative stress) [36], the localization of TMIGD1 at mitochondria of sparsely grown cells may serve to regulate physiological adaptations and signal transduction events that are mediated by ROS. The localization of plasma membrane-resident proteins at mitochondria has been observed for other plasma membrane proteins, including the EGF receptor and the G protein-coupled receptor VLGR1 [37, 38]. On the other hand, the TMIGD1 – SYNJ2BP interaction could also serve to recruit mitochondria to the plasma membrane when cells reach confluency to regulate oxidative stress directly at the membrane [39]. Finally, mitochondria-independent function of the TMIGD1 interaction with SYNJ2BP should also be considered, for example the endocytosis of TMIGD1. The functional relevance of this interaction requires further exploration.

### NHERF1 and NHERF2

Na^+^/H^+^ exchanger regulatory cofactor 1 (NHERF1, a.k.a. Ezrin-binding Phosphoprotein 50, EBP50), and NHERF2 (a.k.a. NHE3 Kinase A Regulatory Protein, E3KARP) are paraloges with a similar size (358 AA vs 337 AS, respectively) and a very similar overall organization consisting of two PDZ domains and an ezrin-binding (EB) region at their C-terminal tail [40]. In polarized epithelial cells, the two proteins localize predominantly to the apical membrane. Through their PDZ domains they interact with the carboxyterminal PBMs of a number of integral membrane proteins, including G protein-coupled receptors (GPCR), receptor tyrosine kinases, ion channels and transporters, and regulate their trafficking and functions [40, 41]. They also interact with various cytoplasmic proteins that are part of signaling pathways including the PI(3)K/AKT, the p38 MAPK, and the Wnt/β-catenin signaling pathway [41]. Through their EB region they interact with ezrin, which is a member of the Ezrin – Radixin – Moesin (ERM) family [42]. ERM proteins can interact with transmembrane proteins, phospholipids, membrane-associated cytoplasmic proteins, and the actin cytoskeleton, and thus link the plasma membrane to the actin cytoskeleton and also organize plasma membrane domains involved in signaling [42]. Of note, NHERF1 but not NHERF2 adopts a dormant state in which the two PDZ domains are inaccessible to ligands [43]. The open conformation enabling ligand binding to its PDZ domains requires the interaction of ezrin with the EB region of NHERF1 [44]. Given the numerous interaction partners of NHERF1/2 scaffolding proteins and their role in various signaling pathways it is not surprising that NHERF proteins have been implicated in various cancers [41]. In particular for NHERF1, a number of studies describe a functional role in cancer cells (summarized in ref [41]). In breast cancer cells, NHERF1 stabilizes the G protein-coupled estrogen receptor GPER1 [45]. In pancreatic cancer cells NHERF1 promotes proliferation and invasiveness by assembling a complex consisting of the chemokine receptor CXCR2 and PLCβ3 [46]. In hepatocellular carcinoma and colorectal carcinoma cell lines, NHERF1 regulates the activity of the Wnt signaling pathway through its association with β-catenin [47, 48].

**Fig. 2:**
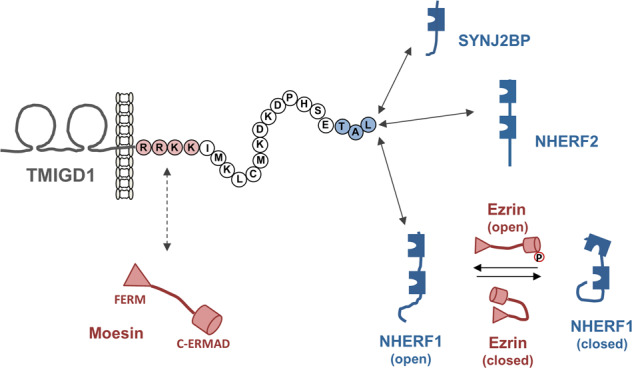
TMIGD1-interacting proteins. Proteins known to interact with TMIGD1. The scaffolding proteins SYNJ2BP/OMP25, NHERF2/E3KARP and NHERF1/EBP50 directly interact with the C-terminal PDZ domain-binding motif (TAL, shown in light blue) of TMIGD1. Note that TMIGD1 interacts with NHERF1 only after active, i.e., T567-phosphorylated, Ezrin interacts with NHERF1 resulting in the open conformation of NHERF1. The FERM domain family member Moesin interacts with TMIGD1 through the juxta-membrane FERM domain-binding motif (RRKK, shown in rose) of TMIGD1.

**Fig. 3:**
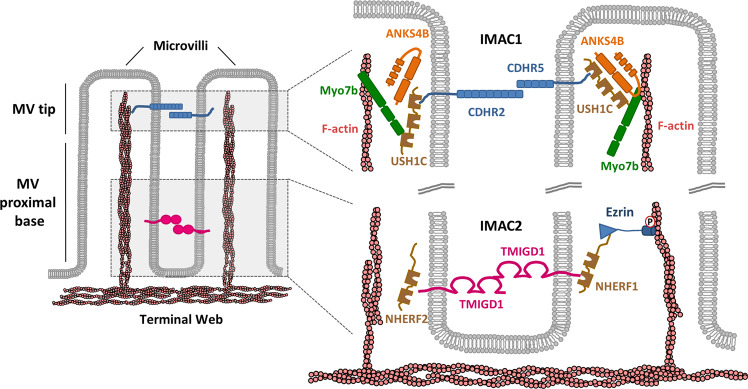
The TMIGD1-based adhesive complex at the proximal base region of microvilli. The apical domain of intestinal epithelial cells is characterized by a brush border consisting of numerous microvilli (MV). Individual microvilli are linked by two distinct intermicrovillar adhesion complexes (IMACs). The IMAC1 is localized at the tips of microvill (MV tip). Adhesion by the IMAC1 is mediated by protocadherins “Cadherin-related family member 2” (CDHR2) and CDHR5, which interact in a trans-heterophilic manner and which are linked to the underlying actin cytoskeleton through the scaffolding proteins USH1 and ANKS4B, and the unconventional myosin MYO7b. The IMAC2 is localized at the proximal base region of microvilli (MV proximal base). Adhesion of the IMAC2 is mediated by trans-homophilic interaction of TMIGD1 molecules, which interact with the cytoplasmic scaffolding proteins NHERF1 and NHERF2. The interaction with NHERF1 requires prior “opening” of the closed conformation by active T567-phosphorylated, active Ezrin. For simplicity, the interactions of TMIGD1 with NHERF1 and NHERF2 are depicted in two separate MV but are expected to occur in the same MV (see ref [21] for details).

TMIGD1 interacts with both NHERF1 and NHERF2 through a mechanism that involves the PBM of TMIGD1 and the PDZ domains of the NHERF proteins [21] (Fig. 2). While the interaction of TMIGD1 with NHERF2 can be demonstrated in vitro in the absence of additional factors, the in vitro interaction of TMIGD1 with NHERF1 requires the presence of Thr567-phosphorylated ezrin, indicating that activated ezrin is necessary to induce the open NHERF1 conformation to allow binding of TMIGD1 to NHERF1, most likely by inducing the open conformation of NHERF1 and thus making the NHERF1 PDZ domains accessible to TMIGD1 (Fig. 2) [21]. This suggests that the interaction between TMIGD1 and NHERF1 most likely is dynamically regulated. Interestingly, and rather uncommon for an adhesion receptor, TMIGD1 is localized at the brush border of intestinal epithelial cells where it is specifically enriched at the proximal base region of microvilli [16, 18, 21]. Since both NHERF1 and NHERF2 are localized in microvilli [49–53], and since ezrin is an important regulator of microvilli dynamics [54, 55], the direct interaction of TMIGD1 with NHERF1 and NHERF2, and its indirect interaction with ezrin through NHERF1 [21], strongly suggest that TMIGD1 exists in a complex with NHERF1, NHERF2 and ezrin in microvilli. Also, in a model system of enterocyte polarization [56], TMIGD1 is recruited to the brush border by either NHERF1 or NHERF2 [21]. Finally, enterocyte-specific inactivation of the *Tmigd1* gene in mice results in a loss of the typically uniform length of microvilli and in blebbing of the microvillar membrane [21]. Based on these observations, the interaction of TMIGD1 with the two NHERF proteins most likely occurs at the brush border of intestinal epithelial cells and serves to regulate the dynamic formation and turnover of microvilli in these cells. The characterization of the association of TMIGD1 with NHERF1 and NHERF2 also identified a second intermicrovillar adhesion complex (IMAC) at the base of microvilli. Another IMAC, which is based on a heterophilic interaction of the two adhesion receptors cadherin-related family member 2 (CDHR2) and CDHR5, has been identified at the distal tips of microvilli [57, 58] (Fig. 3).

### Moesin

As pointed out above, moesin is a member of the ERM family of proteins [42]. These proteins are characterized by a “Four point 1, Ezrin, Radixin, Moesin” (FERM) domain at their amino terminus which can directly bind to phospholipids like phosphatidylinositol (4,5)-bisphosphate (PtdIns(4,5)P2) in the membrane, and an ERM-associating domain (ERMAD) at their carboxy terminus which can bind F-actin. All ERM proteins exist in a dormant state in which the N-terminal FERM domain (also called N-ERMAD domain) interacts with the C-terminal ERMAD domain (allso called C-ERMAD domain) thereby masking the binding sites for phospholipids and F-actin. Their function in linking the actin cytoskeleton to the plasma membrane thus depends on prior unfolding of the closed conformation, which can be regulated by phosphorylation but also by high affinity interactions with ligands. Through their FERM/N-ERMAD domain, ERM family proteins interact with a number of transmembrane receptors including receptor tyrosine kinases as well as with PDZ domain scaffolding proteins [59]. ERM proteins are involved in the organization of specialized membrane domains and in many signaling processes at the cell cortex, in particular in those regulating actin turnover, including cell adhesion, cell migration, or microvillar dynamics [58, 59].

TMIGD1 exists in a complex with moesin through an interaction that involves the FERM domain of moesin and a membrane-proximal motif of positively charged AA in the cytoplasmic domain of TMIGD1 [22] (Fig. 2). Similar clusters of positively charged AA have been found to interact with ERM proteins in binding assays with recombinant proteins in vitro [60], suggesting that this interaction is direct. Since TMIGD1 does not contain a canonical FERM domain-binding motiv (R/K/E-X-X-T-(Y/L)-X-X-A/G) [42, 61], in vitro binding experiments with recombinant proteins will be needed to obtain further information on the nature of the interaction between TMIGD1 and moesin. Interestingly, ectopic expression of TMIGD1 in cultured epithelial cell lines impairs filopodia formation, stabilizes microtubules (MT) and slows down cell migration [22]. The stabilization of MTs by TMIGD1 expression can be reversed by depletion of moesin, which stabilizes MTs at the cell cortex [62], suggesting that the interaction of TMIGD1 with moesin serves to regulate MT turnover and dynamics in migrating cells. Thus, besides its role in regulating microvilli formation and dynamics through its interaction with NHERF1, NHERF2 and ezrin [21], TMIGD1 regulates another highly dynamic process through an interaction with an ERM family protein.

### Cellular functions of TMIGD1

Based on the different subcellular localizations of TMIGD1 and the various direct and indirect interaction partners it is conceivable that TMIGD1 regulates various cellular functions. The molecular mechanisms underlying these cellular functions are largely unexplored and will, therefore, only briefly be discussed. Cell migration: Several studies describe a role of TMIGD1 in cell motility-related processes, such as cell migration, cell invasion and cell spreading [19, 20, 22–24, 63]. These studies are based on observations that ectopically expressed TMIGD1 limits these motility-related processes. In kidney-derived cell lines ectopic TMIGD1 limits the formation of filopodia-like protrusions [22] suggesting the possibility that TMIGD1 regulates the activity of Rho family small GTPases [22, 64]. If TMIGD1 directly affects Rho family GTPase activities, for example by interacting with a RhoGEF or a RhoGAP, has not been demonstrated, yet. As mentioned above, ectopic TMIGD1 stabilizes microtubules suggesting an influence on cell migration by regulating microtubule dynamics [22, 65]. Barrier function: Ectopic expression of TMIGD1 results in an increased barrier function in cultured kidney epithelial cells [24]. This effect is most likely indirect as it is observed in HEK293 cells which do not form typical tight junctions, a structure at the apical region of cell-cell junctions in polarized epithelial cell responsible for the paracellular barrier function [66]. Similar observations in non-polarized epithelial cells have been made with other cell adhesion molcules such as JAM-A and cadherins [67]. Protection from oxidative stress: A protective role of TMIGD1 toward oxidative stress has been observed in kidney epithelial cells and mesothelial cells [23, 24]. This protection is most likely mediated by the inhibition of reactive oxygen species (ROS) generation in mitochondria through an as yet unidentified mechanism [23].

## TMIGD1 expression in cancer and inflammation

### TMIGD1 in colorectal cancer

The first study that identified TMIGD1 as a putative tumor suppressor is based on a systematic comparative transcriptome analysis of normal colonic tissue, precancerous non-polypoid lesions, pre-cancerous polypoid lesions, and colorectal cancer (CRC) lesions [16]. Among several thousand genes that were analyzed the *Tmigd1* gene stood out as its mRNA level was progressively downregulated from normal tissue to non-polypoid lesions to polypoid lesions to colorectal cancer tissues. Also, in a list of the 100 genes that displayed significant downregulation in polypoid lesion vs non-polypoid lesion, TMIGD1 ranked at position two. The mRNA expression data were confirmed at the protein expression level by immunohistochemical analyses of ileal tissue. This study also provided a first characterization of TMIGD1 expression and localization in Caco-2 cells, a human colon-derived cell line, and suggested a differentiation-associated expression of TMIGD1 in intestinal epithelial cells [16]. A RNAseq-based comparison of hyperplastic polyp lesions with colorectal adenoma lesions identified TMIGD1 among the top 10 down-regulated genes in colorectal adenoma [68], confirming a gradual downregulation of TMIGD1 expression during the development of colon cancer. Another analysis using CRC-derived microarrays that were based on 369 carcinoma samples from 9 different datasets of different geographical origin identified TMIGD1 as one among 22 hub genes whose downregulation in CRC samples was most significant [69]. Additional observations describing reduced TMIGD1 expression in CRC samples were made in studies comparing colonic non-adenomatous, non-neoplastic tissue with colonic tumor tissue, with TMIGD1 being among the 11 most significantly downregulated genes in colonic tumor tisse [70]. A study analyzing tissue derived from a rare tumor of the appendix called pseudomyxoma peritonei (PMP) [71] found that TMIGD1 mRNA levels show the second highest downregulation among 34 genes downregulated in PMP tissues [72]. Additional observations based on microarray analyses and on bioinformatic analyses of published datasets further confirmed a downregulation of TMIGD1 expression in human colorectal cancer [19, 63]. In addition, several studies find that low TMIGD1 expression levels in tumors of CRC patients correlate with lower survival rates of the patients [19, 63, 69]. Mice with a constitutive inactivation of the Tmigd1 gene have an altered intestinal tissue morphology and develop intestinal adenoma [19], strongly suggesting that low TMIGD1 levels are not just correlative but rather causative for colorectal cancer development. As evidence for its functional role in cancer development, ectopic expression of TMIGD1 in colorectal cancer cell lines inhibits cell cycle progression at the G2/M transition in vitro, most likely by activating the p38 MAPK pathway (see below for details). In addition, overexpression of TMIGD1 reduces metastastatic spreading of adoptively transferred tumor cell lines in mice [19]. In summary, the frequent and highly significant loss of TMIGD1 expression in CRC tissue, its gradual downregulation during progression from non-polypoid to polypoid to CRC lesions, its role in limiting cell proliferation, and finally its protective function in tumor development and metastasis formation in mice, strongly support a tumor-suppressive function of TMIGD1.

### TMIGD1 in renal cancer

As pointed out before, the kidney is the organ with the second highest expression of TMIGD1 [16, 18–20]. Similar to gastrointestinal malignancies, renal malignancies have been found to be associated with reduced TMIGD1 expression. In the three major renal cell carcinoma (RCC) subtypes, i.e., clear cell renal cell carcinoma (ccRCC), a renal malignancy associated with metastasis and high lethality [73], as well as in papillary renal carcinoma (pRCC) and chromophobe renal carcinoma (chRCC), two non-clear-cell renal carcinoma types with a more favorable outcome [74], TMIGD1 expression is reduced [16, 19, 20]. It should be noted that some contradictory results were obtained in the studies by Cattaneo et al. [16] and the studies by Meyer et al. and De La Cena et al. [19, 20] concerning the relative expression levels of TMIGD1 in ccRCC vs pRCC and chRCC. Importantly, ectopic expression of TMIGD1 in a renal cancer cell line limits tumor formation in vivo after adoptive transfer into nude mice [20]. This function is at least in part based on its ability to activate p38 MAPK signaling. The p38 MAPK pathway is activated by growth factors, environmental stress and inflammatory cytokines, and it co-operates with the second major MAPK pathway, the JNK pathway, in the control of proliferation, differentiation, survival and migration [75]. Importantly, p38α can negatively regulate cell cycle progression both at the G1/S and the G2/M transitions by downregulating cyclins, upregulating cyclin-dependent kinase (CDK) inhibitors, and by phosphorylating the tumor suppressor p53 [75]. The tumor-suppressive activity of ectopic TMIGD1 expression in renal cancer cell lines is associated with activation of p38α, phosphorylation of p53, and upregulation of two CDK inhibitors, CDK inhibitor 1 A (p21CIP1) and CDK inhibitor 1B (p27KIP1) [20], which strongly suggests that the tumor-suppressive activity of TMIGD1 is based on its ability to activate the p38 MAPK signaling pathway. Similar findings were obtained in colorectal cancer cells [19], suggesting that the activation of the p38α MAPK pathway is a major molecular mechanism through which TMIGD1 suppresses cellular transformation.

### TMIGD1 expression in the inflamed intestine

A downregulation of TMIGD1 in inflammatory conditions has been identified in a systematic transcriptomic analysis of mRNA expression levels in inflamed tissue vs uninflamed tissue in patients suffering from Crohns Disease (CD), which is a subtype of inflammatory bowel disease (IBD) characterized by chronic intestinal inflammation [76]. The *TMIGD1* gene stood out as its expression was reduced by a fold change (FC) value −2.74 (Log base 2) with the lowest statistical error rate in an analysis of 19 CD patients [18], which is suggestive of a very strong and robust downregulation in inflamed tissue. In an experimental study of dextran sodium sulfate (DSS)-induced colitis in mice, TMIGD1 was found to be downregulated by a FC value −2.09 (Log base 2) [77]. Also, a study using a mouse model of chronic inflammation-induced CRC identified TMIGD1 among the most significantly downregulated genes in inflamed intestinal tissue [78]. Although the evidence suggesting a role of TMIGD1 during inflammation is still limited, these findings further support a function of TMIGD1 in tissue homeostasis. Since inflammation is a known risk factor for colorectal cancer [79] it is conceivable that an inflammation-induced downregulation of TMIGD1 is a causative factor contributing to the development of colorectal cancer.

## Regulation of TMIGD1 gene expression: promoter methylation, transcription factors and miRNAs

The expression of TMIGD1 is observed predominantly in tissues of the gastrointestinal tract and in the kidney (https://www.proteinatlas.org/ENSG00000182271-TMIGD1/tissue). As pointed out in the previous section, TMIGD1 is frequently and highly significantly downregulated in cancerous tissue derived from the gastrointestinal tract and from kidney. Despite its tissue-specific expression and its apparent function as tumor suppressor, the mechanisms regulating TMIGD1 gene expression are largely unknown. Some studies provide a first insight into the regulation of TMIGD1 expression.

The human TMIGD1 promoter region lacks canonical CpG islands [16]. However, methylation of CpG sites in the promoter region of the murine *Tmigd1* gene that are located outside of CpG islands have been identified [80]. Significant methlyation of these sites has been found in muscle and liver tissues both before and after adulthood. A DNA methylation analysis based on human disease methylation database (DiseaseMeth version 2.0) [81] revealed that the TMIGD1 gene is in fact methlyated in colonic and rectal carcinoma [68], suggesting that methylation of the TMIGD1 promoter contributes to the silencing of the TMIGD1 gene during development of colon carcinoma.

**Fig. 4:**
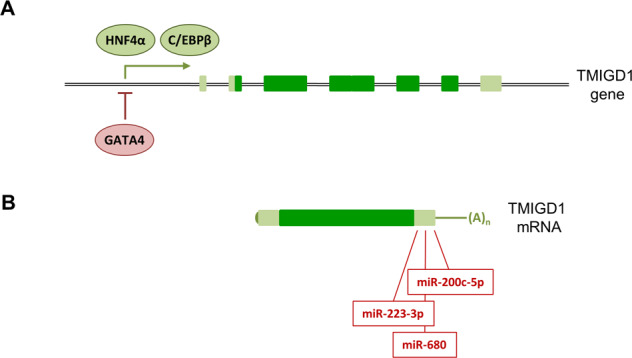
Regulation of TMIGD1 gene expression. **A** TMIGD1 gene and transcription factors. Exons are indicated by dark green (coding regions) and light green (non-coding regions) bars. Transcription factors which regulate TMIGD1 gene expression are indicated in oval green (activating) and rosé (inhibiting) symbols. **B** Posttranscriptional regulation of TMIGD1 mRNA by micro RNAs. The relative positions of seed sequences of miRNAs miR-223-3p, miR-680 and miR220c-5p are shown. All three miRNAs are predicted to target the 3′-UTR of the TMIGD1 mRNA. A functional interaction with the TMIGD1 mRNA has been demonstrated for miR220c-5p.

In silico analyses have identified several putative transcription factor binding sites (TFBSs) around the transcription start site (Fig. 4), including a binding site for hepatocyte nuclear factor-4α (HNF4α) [16], a transcription factor which is downregulated in precancerous lesions of colorectal tissues [16]. The TMIGD1 promoter contains a functional HNF4α binding site as identified in a ChIP-chip study [82], and, based on microarray analyses, HNF4α regulates TMIGD1 expression [83]. Intriguingly, the expression of HNF4α is repressed by TGFβ [83], and importantly, TGFβ also represses TMIGD1, which can be reversed by ectopic expression of HNF4α [83], suggesting that TGFβ regulates TMIGD1 expression through HNF4α. TGFβ is a potent inducer of epithelial-to-mesenchymal transition (EMT), a process that contributes to invasive cell behavior and to a metastatic phenotype during cancer development [4, 84]. As a further support for a role in regulating TMIGD1, which is expressed by well-differentiated enterocytes but not in intestinal crypts [16, 18], HNF4α has been found to be central during enterocyte differentiation [85]. Alltogether, these findings identify HNF4α as a strong candidate transcription factor regulating TMIGD1 expression in intestinal tissues. Together with the observation of TMIGD1 downregulation in colorectal cancer, they also suggest that TGFβ-triggered downregulation of TMIGD1 via HNF4α is part of the TGFβ-induced EMT programme.

A second putative TMIGD1-regulating transcription factor is GATA-binding factor 4 (GATA4). Along the intestinal epithelium, GATA4 is expressed in enterocytes of the duodenum and jejunum but is absent in enterocytes of the ileum [86]. When GATA4 is ectopically expressed in the ileum using a conditional knock-in approach, the gene expression pattern shifts from a ileum-specific profile to a jejunum- and duodenum-specific profile [87]. TMIGD1 is among several genes whose expression is suppressed upon ectopic GATA4 expression in the ileum and, vice versa, whose expression is increased in the jejunum of GATA4 knock-out mice [87], which suggests that GATA4 represses TMIGD1 gene transcription. In line with a direct role of GATA4 in regulating TMIGD1, GATA4 binding sites have been identified in the murine TMIGD1 gene promoter [87]. Thus, GATA4 and HNF4α appear as important transcription factors responsible for TMIGD1 gene expression in the intestinal tract.

A third transcription factor which regulates TMIGD1 gene expression is CCAAT/enhancer-binding protein β (C/EBPβ, a.k.a. Liver activator protein, LAP). The human TMIGD1 gene contains several putative C/EBP binding sites in its promoter, and C/EBPβ interacts with the TMIGD1 promoter as shown by electrophoretic mobility shift assays (EMSA) [20]. Similar to the expression levels of TMIGD1, the levels of C/EBPβ are low in renal cancer as well as in kidney cancer-derived cell lines [20]. Importantly, ectopic expression of C/EBPβ in a kidney cancer-derived cell line results in a strong upregulation of TMIGD1 expression [20]. These studies make a strong point for C/EBPβ as a major transcription factor regulating TMIGD1 gene expression in the kidney. Of note, C/EBPβ expression is regulated by the p38 MAPK pathway [75], which—as pointed out before—is activated by TMIGD1 [20], suggesting that TMIGD1 expression is regulated by a positive feed-back regulatory loop.

On the basis of (comparative) microarray profiling, several microRNAs (miRNAs) that could target TMIGD1 have been identified (Fig. 4). In a cell culture model of mouse myoblast differentiation, a downregulation of miR-200c-5p during differentiation has been observed [88]. The murine Tmigd1 mRNA contains a miR-200c-5p seed sequence in the 3′-UTR, and, importantly, a luciferase reporter system showed a functional interaction of miR-200c-5p with the 3′-UTR of the murine Tmigd1 mRNA in HEK293T cells [88], making miR-200c-5p a strong candidate for the regulation of TMIGD1 mRNA stability [88]. Two other candidates are miR-223-3p and miR-680. Both miRNAs are predicted to target TMIGD1 acc. to the “miRBase” microRNA database (https://www.mirbase.org/), with miR-223-3p being predicted by five different miRNA target prediction algorithms [89]. The expression of both miRNAs is markedly increased (FC values for miR-223-3p and miR-680: 3.42 and 2.83, Log base 2 each) in inflamed large intestine, in which the expression of Tmigd1 is markedly downregulated (FC value −2.74, Log base 2) [18, 77]. Although the interaction of miR-223-3p and miR-680 with the TMIGD1 mRNA still have to be experimentally tested, together with miR-220c-5p they represent potential candidate miRNAs involved in the regulation of TMIGD1 expression.

## Summary and conclusions

TMIGD1 is a cell adhesion receptor that has drawn attention after the discovery that its expression is progressively downregulated during the development of colorectal cancer in humans [16]. Meanwhile, a number of studies have confirmed a highly significant downregulation not only in colorectal cancer but also in renal cancer, strongly supporting a tumor-suppressive function of TMIGD1 and suggesting that TMIGD1 expression may be used as a prognostic marker [19, 63, 69]. Also, the molecular biology underlying its functions is beginning to be understood. For example, several binding partners have been identified, which suggests that TMIGD1 has pleiotropic functions in the cell [21, 22, 25]. In addition, intracellular signaling pathways that are activated by TMIGD1 were identified, which has provided the first insights into the molecular mechanisms underlying its tumor-suppressive activity [19, 20]. However, many question will have to be addressed in the future. At the tissue level it will be important to understand the function of TMIGD1 in the regulation of cell differentiation in the intestine and in the kidney. It will also be important to understand the influence of inflammation on TMIGD1 expression. At the cellular level it will be important to understand how TMIGD1 as an adhesion receptor regulates mitochondrial function, and how it regulates cellular functions related to cell motility. Clarifying its function at the molecular and cellular level will be important to understand the tumor-suppressive function of cell-cell adhesion.

## Data Availability

NA (no original data were used in this paper).
